# Analysis of mutations in the E6 oncogene of human papillomavirus 16 in cervical cancer isolates from Moroccan women

**DOI:** 10.1186/1471-2334-13-378

**Published:** 2013-08-16

**Authors:** Zineb Qmichou, Meriem Khyatti, Mohamed Berraho, My Mustapha Ennaji, Laila Benbacer, Chakib Nejjari, Noureddine Benjaafar, Abdellatif Benider, Mohammed Attaleb, Mohammed El Mzibri

**Affiliations:** 1 Unité de Biologie et Recherche Médicale, Centre National de l'Energie, des Sciences et des Techniques Nucléaires, (CNESTEN), BP 1382 RP, 10001 Rabat, Morocco; 2 Laboratoiore de Microbiologie, Hygiène et Virologie, FST Mohammedia, Mohammedia, Morocco; 3 Laboratoire d’Oncovirologie. Institut Pasteur du Maroc, Casablanca, Morocco; 4 Laboratoire d'Epidémiologie, Recherche clinique et Santé Communautaire, Faculté de Médecine et de Pharmacie, Fès, Morocco; 5 Service de radiothérapie, Institut National d'Oncologie de Rabat, Rabat, Morocco; 6 Centre Mohammed VI pour le Traitement des Cancers, Casablanca, Morocco

**Keywords:** Cervical cancer, Human papillomavirus 16, Variants, E6, Morocco

## Abstract

**Background:**

Worldwide, cervical cancer is the second most common cancer in women. High-risk human papillomavirus (HPV) play a crucial role in the etiology of cervical cancer and the most prevalent genotype is HPV16. HPV 16 intratypic variants have been reported to differ in their prevalence, biological and biochemical properties. The present study was designed to analyze and identify HPV type 16 E6 variants among patients with cervical cancer in Morocco.

**Methods:**

A total of 103 HPV16 positive samples were isolated from 129 cervical cancer cases, and variant status was subsequently determined by DNA sequencing of the E6 gene.

**Results:**

Isolates from patients were grouped into the European (E), African (Af) and North-American (NA1) phylogenetic clusters with a high prevalence of E lineage (58.3%). The Af and NA1 variants were detected in 31.1% and 11.6% of the HPV16 positive specimens, respectively, whereas, only 3% of cases were prototype E350T. No European-Asian (EA), Asian (As) or Asian-American (AA) variants were observed in our HPV16-positive specimens. At the amino acid level, the most prevalent non-synonymous variants were L83V (T350G), H78Y (C335T), E113D (A442C), Q14D (C143G/G145T) and R10I (G132T), and were observed respectively in 65%, 41.8%, 38.8%, 30.1% and 23.3% of total samples.

Moreover, HPV16 European variants were mostly identified in younger women at early clinical diagnosis stages. Whereas, HPV16 Af variants were most likely associated with cervical cancer development in older women with pronounced aggressiveness.

**Conclusion:**

This study suggests a predominance of E lineage strains among Moroccan HPV 16 isolates and raises the possibility that HPV16 variants have a preferential role in progression to malignancy and could be associated with the more aggressive nature of cervical cancer.

## Background

Cervical cancer is the second most predominant cancer worldwide, and more than 85% of the global burden occurs in developing countries, where it accounts for 13% of all female cancer [1]. In Morocco, as it is the case in the other North African countries, cervical cancer is the second most common cancer among women and its incidence is the highest in this region with an age-standardized incidence rate (ASR) of 13.5 per 100 000 women [2]. Persistent infection with high risk Human papillomavirus (HPV) is the main aetiological factor in the development of cervical cancer. Globally, HPV16 is the most frequent HPV type found in cervical cancer and is identified in approximately 65% of cases [3]. In Morocco, our previous reports have shown that the HPV16 is also the most frequent type of HPV found in cervical cancer patients [4-6].

Given that the prevalence of cervical cancer varies in different regions and countries, a number of studies have addressed the possible association of HPV16 variant status with the risk for progression to malignancy. HPV variants are defined as isolates with primary DNA sequence differences that total no more than 2% of the L1 open reading frame (ORF) of the prototype sequence [7]. The sequence variations of the HPV16E6 ORF have been found to correctly classify the HPV16-variants [8]. Therefore, most studies on HPV16 variants have focused on E6, the major transforming protein that inhibit apoptosis and promote proliferation and their association to cancer aggressiveness [9-11]. Worldwide, variants of the HPV16 genome in cervical carcinoma, precancerous lesions and normal or inflammatory cervical cases, from different geographical areas have been classified into phylogenetic lineages as European (E), Asian (As), Asian-American (AA), African -1 and -2 (Af1 and Af2), and North American1 (NA1) [8,12,13]. Overall, multiple studies have shown that HPV16 variants differ in risk for progression to high grade intraepithelial lesions [14-16], in their association with the development of cervical cancer [15,17-19], viral persistence [17,20-26] and the frequency of recurrence of cervical disease [15].

Many authors confirm that distribution of HPV variants is related to geographic or race distribution [27]. However, although numerous studies on HPV16 variants in cervical cancer have been carried out so far worldwide, only one HPV variant research in patients from Tunisia has been performed in North Africa [28]. To our knowledge, nothing is known concerning HPV variants in patients from Morocco, where there is a significantly high occurrence of this tumor. In the present study entirely done in Morocco, we have examined whether naturally occurring sequence variations in the HPV16 E6 gene exist in cervical cancer from Moroccan subjects with HPV 16 infection. The identification of more persistent or oncogenic HPV16 variants would be of great interest in the global strategy in the development and implementation of improved therapeutic vaccines against HPV.

Furthermore, HPV sequence variations studies can be used in epidemiological studies as a marker to track the spread of the virus in contact networks through populations.

## Methods

### Clinical specimens

One hundred twenty nine fresh frozen uterine cervix biopsies were recruited for the variant analysis in the present study. The tumor samples were from biopsies collected for the routine diagnosis of cervical cancer from Gynaecology department of the National Institute of Oncology in Rabat and Oncology Centre Mohammed VI pour le Traitement des Cancers, CHU Ibn Rochd in Casablanca, that receive patients from all regions of Morocco. The mean age of patients was 52 ± 12 years, with extreme ages at 28 and 80. Each sample was divided into two portions: one portion was put in neutral buffered formalin and processed for routine histopathological examination; the other portion was stored at −80°C immediately after surgical removal, for HPV genotyping. Protocols were approved by Ethical Review Committees of the Faculty of Medicine of Fez and the Hassan-II University Hospital, and written informed consent was obtained from each study subject.

### Cell lines

CaSki and SiHa cervical cancer cell lines were obtained from the American Type Culture Collection (ATCC) and used as positive controls. These cell lines were maintained in Dulbecco’s modified Eagle’s medium (DMEM) supplemented with 10% fetal calf serum (FCS).

### Pathology

Samples were fixed in 10% buffered-formaldehyde. Pathology diagnosis was made using the routine hematoxylin***-***eosin stain on 5 μm paraffin sections. Histopathological results of cervical cancer lesions were classified according to the International Federation of Gynaecology and Obstetrics (FIGO).

### DNA extraction

Genomic DNA was extracted from fresh frozen tissue and cervical cancer cell lines using a standard technique of digestion with proteinase K in the presence of sodium dodecyl sulfate (SDS) at 37°C overnight, followed by phenol/chloroform purification [29]. The tissue DNA was precipitated with 2/5 volumes of 7.4 M ammonium acetate and 2 volumes of 100% ethanol, followed by incubation at −20°C and centrifugation at top speed (13000 relative centrifugal force). DNA was then resuspended in 25–30 μL of sterile distilled water and stored at −20°C until use. In order to evaluate the efficiency of DNA extraction, all samples were amplified by PCR using PC04 and GH20 primers specific for human β-globin gene (Table 1).

**Table 1:** List of primers used for PCR amplification and DNA sequencing

	**Primer**	**Fragment generated size**	**Sequence**	**Tm (°C)**
β-globin	PC04	268 bp	5′-CAACTTCATCCACGTTCACC-3′	54.5
	GH20		5′-GAAGAGCCAAGGACAGGTAC-3′	
HPV detection & genotyping	MY09	450 bp	5′-CGT CCM ARR GGA WAC TGATC- 3′	55
	MY11		5′-GCM CAG GGW CAT AAY AAT GG-3′	
	GP5+	150 pb	5′-TTTGTTACTGTGGTAGATACTAC-3′	55
	GP6+		5′-CTTATACTAAATGTCAAATAAAAA-3′	
HPV16 E6 variants	F	524 bp	5′-CGAAACCGGTTAGTATAA-3′	55
	R		5′-GTATCTCCATGCATGATT-3′	

F: Forward primer; R: Reverse primer

### HPV genotyping

HPV detection and genotyping of cervical cancer cases were performed using a nested PCR, with the consensus MY09/11 and GP5+/6+ primers (Table 1), and direct DNA sequencing [30]. All specimens were first subjected to PCR amplification with HPV consensus primers MY09/11 [31]. For nested PCR, 2 μL of the MY09/11 PCR products was used as a template for PCR amplification using GP5+/6+ general primers [30,32]. For every reaction, a negative control, in which DNA template was omitted from the amplification mixture, and a positive control, with DNA extracted from SiHa and Caski cell lines, were included. PCR products were analyzed by electrophoresis on 2% agarose gels followed by staining with ethidium bromide (10 mg/mL).

For DNA sequencing, the PCR products were purified by the ExoSaP-IT clean up system (USB, USA) and sequenced directly on an ABI 3130XL DNA analyzer (Applied Biosystems, Foster city, CA, USA), using GP6+ primer as the sequencing primer and BigDye® Terminator v3.1 Cycle Sequencing Kit (AppliedBiosystems, Foster city, CA, USA), according to manufacturer’s protocol. For each sample, PCR amplification and DNA sequencing were performed twice. Nucleotides sequences were aligned and compared with those of known HPV types available through GenBank by using the online BLAST 2.0 software server (http://www.ncbi.nih.gov/blast). In accordance with established guidelines, a nucleotide sequence was assigned to an HPV type if it corresponded with a known HPV genotype by >90% [33].

### Determination of HPV16 E6 variants

Samples with a single HPV16 genotype were selected for E6 variants investigation. Determination of HPV16 E6 variants was performed by E6 specific PCR, using primers flanking the encoding region of HPV16 E6 gene (nt 52–575) (Table 1), and DNA sequencing.

PCR amplifications were performed according to Lizano et al. [34]. For every reaction, positive controls using DNA extracted from SiHa and Caski cell lines, as well as a negative control without template DNA, were included. PCR products were tested on an ethidium bromide stained 2% agarose gel. PCR products were purified and sequenced with the same primers as those used for the amplification [34]. The HPV16 E6 sequences obtained were aligned with those of European prototypes of HPV16 sequence (HPV16-R) (GenBank accession numbers: K02718, NC_001526), available through the GenBank database (NCBI, National Institute of health, Bethesda, MD, USA), using the BLAST 2.0 software server (http://www.ncbi.nih.gov/BLAST/), MEGA v4.0 and SeqScape v2.6 (Applied Biosystems, Foster city, USA) programs.

### Statistical analysis

The HPV16 variants according to other clinical features were compared using a Fisher’s exact test using the MedCalc statistical software (http://www.medcalc.org). The statistical relationship was considered as significant if the derived p value was < 0.05.

## Results

### Histopathological data

The pathological analysis revealed the predominance of Squamous Cell Carcinoma (SCC) that represents 96.89% of cases (125/129). The remaining 4 cases (3.1%) were diagnosed as adenocarcinoma. According to stage, to the most important percentages were found in stage II and III representing 48.8% (61 cases) and 38.4% (48 cases), respectively. Moreover, 14 cases were classified as stage I (11.2%), whereas only 2 cases (1.6%) were classified as stage IV.

### Subtype distribution of HPV DNA

The presence of amplifiable DNA was confirmed for all 129 cases by PCR based-technique using primers for a fragment of β-globin gene and therefore all DNA samples were adequate for further analysis. Molecular detection of HPV DNA, using nested PCR amplification of a conserved region of the HPV L1 gene DNA with the consensus MY09/11 and GP5^+^/6^+^primers, revealed the presence of viral DNA in 91.47% (115 of 129 SCC and 3 of 4 adenocarcinoma).

In this study, we have identified 5 carcinogenic HPV genotypes: 16, 18, 31, 33 and 35. The most frequently found was HPV16, detected in 87.29% of all positive samples (103 cases), followed by HPV 18, present in 5.08% of samples (6 cases), HPV 31 in 2.54% of samples (3 cases), HPV 33 in 1.69% of samples (2 cases) and HPV 35 in 1 specimen (0.85% of cases). Undetermined HPV genotypes were identified in 3SCC cases.

### Sequence variations of HPV16 E6 gene

The 103 HPV16 single infected specimens were selected for E6 intratypic variation analysis. Overall, DNA sequences were compared to the HPV16 reference sequence (Accession Number: K02718, NC_001526). Accordingly, HPV16-positive specimens were classified into E, Af1, Af2 and NA branches (Table 2).

**Table 2:** Characterization of HPV16 E6 variants and their lineage classification

	**HPV 16 variants**		**N**	**T109C**	**A131G**	**G132T**	**C143G**	**G145T**	**C285G**	**T286A**	**A289G**	**T295G**	**T325C**	**C335T**	**T350G**	**A403G**	**A442C**	**Predicted substitution**
	**HPV16 Reference**			**T**	**A**	**G**	**C**	**G**	**C**	**T**	**A**	**T**	**T**	**C**	**T**	**A**	**A**	
**Lineage**	**Sub-lineage**	**Class/sub-class**																
**EUR**	Ep	E-T350	3	-	-	-	-	-	-	-	-	-	-	-	-	-	-	
	E-350G	E-G350	16	-	-	-	-	-	-	-	-	-	-	-	G	-	-	L83V
		E-C442/G350	40	-	-	-	-	-	-	-	-	-	-	-	G	-	C	L83V/E113D
		E-G350/T145	1	-	-	-	-	T	-	-	-	-	-	-	G	-	-	Q14H/L83V
**AFR**	Af1	Af1-d/G295	5	-	G	-	G	T	-	a	g	G	-	T	G		-	R10G/Q14D/D64E/H78Y/L83V
		Af1-d/G295/C325	2	-	G	-	G	T	-	a	g	G	c	T	G	-	-	R10G/Q14D/D64E/H78Y/L83V
	Af2	Af2-a/C109/G403	13	c	-	T	G	T	-	a	g	-	-	T	-	g	-	R10I/Q14D/H78Y
		Af2-a/G285	1	-	-	T	G	T	G	a	g	-	-	T	-	g	-	R10I/Q14D/A61G/H78Y
		Af2a/r	10	-	-	T	G	T	-	a	g	-	-	T	-	g	-	R10I/Q14D/H78Y
**NA**	NA1	NA1	9	-	-	-	-	-	-	a	g	-	-	T	-	g	-	H78Y
		NA1	2	-	-	-	-	-	-	a	g	-	-	T	G	-	-	H78Y/L83V
		NA1-b/r	1	-	-	-	-	T	-	a	g	-	-	T	G	-	-	Q14D/H78Y/L83V
N*				13	7	24	31	33	1	43	43	7	2	43	67	33	40	

Sequence alterations relative to the E6 open reading frame (ORF) of the reference HPV16 sequence [35]. For subclasses, the number and the letter represent the nucleotide change at that position. Lower case represents a nucleotide change at that position without an amino acid change. Capital letters indicate variants with an amino acid change. In the ‘Predicted substitution’ column, the letter preceding the amino acid position refers to the reference HPV16 sequence and the letter after it refer to the substitution. Dashes indicate no variation. * Total cases harboring the point mutation. EUR: European lineage, AFR: African lineage, NA: Nord American lineage.

By comparison with the prototype sequence of HPV16 E6, it was found that only 2.9% of samples (3 out 103) revealed the prototype sequence, while 97.1% of samples (100 out 103) showed at least one specific nucleotide variation in the HPV16 E6 region (Table 2). Moreover, in all analyzed specimens, there was no evidence of premature stop codons, bases insertions or deletions.

Overall, 9 variants with predicted E6 amino acid changes were identified (Table 2). The most prevalent non-synonymous variants were L83V (T350G), H78Y (C335T), E113D (A442C) and Q14D (C143G/G145T) and were observed respectively in 65%, 41.8%, 38.8% and 30.1% of total samples. The other non-synonymous variants were R10I (G132T), R10G (A131G), D64E (T295G), Q14H (G145T) and A61G (C285G) and were seen in 23.3%, 6.8%, 6.8%, 1.9% and 1% of cases respectively.

A total of 12 different intratypic variants were identified among the 103 HPV16 specimens isolated from Moroccan women. These intratypic variants are grouped into three different variant classes, including European (E), African (Af) and the North-American (NA1) variant classes. The European variants HPV16 were the most frequently detected isolates in our population and represent 58.3% of total HPV16 isolates (n=60), followed by Af characterized in 30.1% of cases (n=31). Whereas the NA variants were found only in 11.6% of cases (n=12) (Table 2). In the European class, two sub-classes prevail, E-350G/442C identified in 40 specimens (38.8%) and E-350G identified in 16 specimens (15.5%). The common no-E variants are represented by Af2, NA1 and Af1 sub-classes, which are identified in 24 (23.3%), 12 (11.6%) and 7 (6.8%) cases, respectively. Asian (As) and Asian-American (AA) HPV16 variants were not detected in the present study.

The distribution of the different E6 variations in the HPV16 positive samples with respect to FIGO staging of cervical neoplasia is reported in Table 3. E class variants, the most common intratypes, were mainly associated with cancers detected in early clinical stages. The majority of cervical neoplasia with HPV16 E variant lesions presented at stage I and II with 75% (9/12) and 60% (30/50) respectively. Conversely, Af variants seem to be most likely detected in advanced cancer stages, there were detected in 37.5% (15/40) of cases at stage III and only in 16.67% (2/12) and 28% (14/50) of cases at stages I and II respectively. NA1 variants were detected with similar frequencies in early as well as advanced stages. However, no significant statistical difference between HPV variant and stage (early I and II, advanced III and IV) of cancer was detected (*p* > 0.05).

**Table 3:** Distribution of HPV16 E6 variants according to clinical stage

**Clinical stage**	**N**	**E**				**Af**		**NA**
		**E350G**	**E350G/442C**	**E-G350/G145**	**Ep**	**Af1**	**Af2**	**NA1**
I	12	2	7	-	-	1	1	1
		9				2		
II	50	10	18	1	1	2	12	6
		30				14		
III	40	4	14	-	2	4	11	5
		20				15		
IV	1	-	1	-	-	-	-	-
		1						
**Subtotal**		16	40	1	3	7	24	12
**Total**	**103**	**60**				**31**		**12**

The distribution of HPV16 E6 variants according to patients’ age was also assessed and is reported in Table 4. Results showed an overrepresentation of Af class in the age group over 45 years old whereas E and NA variants were most identified in the age group under 45 years. Moreover, among women under 45 years, E350G/442C is the most predominant variant with 48.15% of cases (13/27). However, in women over 45 years, two variants prevail: E350G/442C and Af2 which are present in 35.53% (27/76) and 25% (19/76) respectively, statistical analyses were significant for only HPV16 E and Af variants related to patients’ age (*p* < 0.0001).

**Table 4:** Distribution of HPV16 E6 variants according to patients’ age

**Patients’ age**	**N**	**E**				**Af**		**NA**
		**E350G**	**E350G/442C**	**E-G350/G145**	**Ep**	**Af1**	**Af2**	**NA1**
≤ 45 years	27	4	13	-	-	-	5	5
		17				5		5
> 45 years	76	12	27	1	3	7	19	7
		43				26		7
**Subtotal**		16	40	1	3	7	24	12
**Total**	**103**	**60**				**31**		**12**

Correlation between HPV16 E6 variants and malignant phenotype showed that E variants are mostly associated with high degree of differentiation, moderately and well differentiated carcinoma, whereas Af variants are more frequently found in poorly differentiated carcinoma (Table 5).

**Table 5:** Correlation between malignant phenotype and HPV16 E6 variants

**Differentiation**	**N**	**E**				**Af**		**NA**
		**E350G**	**E350G/442C**	**E-G350/G145**	**Ep**	**Af1**	**Af2**	**NA1**
Poorly	17	3	4	-	-	1	7	2
		7				8		
Moderately	39	5	18	1	2	4	7	2
		26				11		
Well	31	6	13	-	-	1	6	5
		19				7		
Unknown	16	2	5	-	1	1	4	3
		8	5					
**Subtotal**		16	40	1	3	7	24	12
**Total**	**103**	**60**				**31**		**12**

## Discussion

The present study, entirely realized in Morocco, highlighted the distribution patterns of intratypic variants of HPV16 among women with cervical cancer. A total of 129 samples of cervical cancer cases were collected. Molecular detection of HPV, using nested PCR amplification of a conserved region of the HPV L1 gene with the consensus MY09/11 and GP5+/6+ primers, revealed the presence of viral DNA in 91.47% of cases (118/129). We identified 5 carcinogenic HPV genotypes (16, 18, 31, 33 and 35). Globally, HPV prevalence and distribution is in concordance with previously reported data in Morocco [4,6], and tally with the overall world distribution of HPV types in cervical cancer [36,37]. Moreover, HPV16 was the HPV genotype most frequently found in our study population, with 87.29% of all positive samples, which is in agreement with the worldwide reported data [3,18,37,38]. However, previous studies conducted in Morocco have reported lower prevalence of HPV16.

Lalaoui *et al*. [4] have found HPV 16 in 49% of HPV positive cases, whereas in the study of Meftah El Khair *et al.*[31], HPV16 was found in 71% of HPV positive cases, including mono-infected (37%) and co-infected (34%) cases. This difference may be related to the sampling bias but also could be due to the molecular technique used for the HPV genotyping. In the previous studies, HPV genotyping was realized by combining consensus PCR and dot blot hybridization with specific probes. However, in our study, the HPV genotyping was performed by DNA sequencing of the hyper-variable region in L1 fragment which is reported to be more accurate and give a high sensitivity [30,33].

To examine the prevalence of naturally occurring sequence variations in the HPV16 E6 gene in cervical cancer from Moroccan subjects, all HPV16 positive samples (n=103) were subjected to intratypic variant analysis by nucleotide sequencing of the E6 gene and comparison with the prototype sequence of HPV16 E6.

Of all HPV16 positive samples, only 3 cases exhibited a prototype E6 gene sequence and 97.1% of cases (100/103) showed at least one specific nucleotide variation in the HPV16 E6 region. This finding is in agreement with published results reporting that approximately 90% of the E6 genes in invasive cervical cancer contained variations [39,40].

It is widely accepted that the distribution of HPV variants is related to geographic or race distribution [21,27] and therefore, owing to the geographic location of Morocco in the North Africa, we expected the predominance of African variants. Unexpectedly, however, the E6 gene sequencing analyses highlighted that the most frequently HPV16 variants detected in our population belonged to the European lineage (58.3%), whereas the African lineage was detected only in 30.1% of cases. The NA1 lineage was less represented and limited to 11.6% of cases. The frequent circulation of the European lineage in Morocco most likely reflects the immigration flow that characterized this region for a long time in the past. Morocco, due to its geographic position, is recognized as a border region between Europe and Africa, and therefore was a corner stone in all exchanges between the two continents. Authors have already reported that HPV strains could be introduced as a result of migration leading to the spread of the molecular variants of HPV from one continent to another [41]. Krennhrubec *et al.*[28] have reported the same results in Tunisia, a neighbor country in North Africa, with 64% of HPV16 infections being of European lineage.

HPV16 European variant is a highly spread variant around the world, a meta-analysis conducted on HPV16 positive tumors samples collected globally from different ethnical group reported that European lineage was the most predominant lineage and was well distributed among the different geographical regions. This widespread presence suggests biological relevance of this variant [42].

A large number of HPV16 variants circulating in our population belong to African branches (30.1% of cases). The association of Af variants with an increased risk of cervical cancer development is controversial. Some authors have reported a greater association between infection with HPV16 Af variants and an increased risk of cervical carcinogenesis [15,43,44]. However, Tu *et al.*[45] have reported no evidence that Af variants could be associated with an increased risk of neoplasia. Within the Af lineage, the two main subgroups, Af1 and Af2 were detected in 7 (6.8%) and 24 (23.3%) cases respectively. These results are in contrast to previous studies that reported the predominance of Af1 variants, among the Af lineage, in North Africa [12,42].

Interestingly, we have detected NA1 lineage in 11.6% of our patients’ population. The occurrence of NA1 classes, which is a derivative of the AA and Af2 variants [12], is difficult to explain. The NA1 lineage, identified in 1990s [12], was recently found to be particularly frequent in samples from North Africa [42]. We believe that NA1 lineage could be introduced recently in Morocco and spread in the population. That could explain the lower frequency of this lineage in the study population.

According to clinico-pathological data, HPV16 European variants (350G alone or along with other mutations) were mostly identified in younger women at early clinical stages (I and II) at diagnosis. However, most of the cancer patients, harboring the HPV16 Af variants, were older (over 45 years) and exhibited a pronounced aggressiveness of the disease, as the majority of cases were at advanced stage (III) with poorly differentiated carcinoma. These results raise the possibility that HPV16 variants have a preferential role in progression to malignancy and could be associated with the more aggressive nature of cervical cancer. Similar results were obtained in Tunisia reporting a higher proportion of non-European HPV16 variants in advanced stage (III). In contrast, the non-E variants were more frequently detected at earlier average age at diagnosis than European variants [28]. This difference could not be due to genetic diversity of the host, as the two populations are very closely related, but could be due to a sampling bias and biological behavior of the host.

Most nucleotide changes reported in our study have been previously described and some of them are of particular interest and may have considerable biological impact [10,46]. The most prevalent non-synonymous variants were L83V (T350G), H78Y (C335T), E113D (A442C), Q14D (C143G/G145T) and R10I (G132T), and were observed respectively in 65%, 41.8%, 37.9%, 30.1% and 23.3%of total samples. Against the above background of higher pathogenicity associated with the L83V variant, the finding of our study reiterates the same. Indeed, the most frequently observed mutation in our study was L83V which is detected in 65% of cases (67/103), either alone (15.5% of cases) or along with other mutations (49.5% of cases). This mutation is the most commonly found in HPV16 E6 gene from cervical invasive cancers [44], and has been linked to an increased risk for cervical disease progression [20]. Many authors addressed a geographic variation of the oncogenicity of this variant [22,39,47]. In some European populations, HPV16 E350G variant was strongly associated with the oncogenicity and persistency of HPV16 infection [20,39]. Moreover, HPV16 E350G variant was found to display more efficient degradation of Bax (protein regulated by p53) and binding to E6-BP (E6 binding protein) and decreased binding to human discs large protein (hDlg) [48]. This variant appears to have surpassed the E6 prototype in enhancing the mitogen activated protein kinase (MAPK) signaling evolved in oncogenic ras-mediated transformation and in the cooperative transformation with the deregulated Notch 1 pathway [49]. Also, this L83V polymorphism appears to interact with natural human variations in the p53 genes (in particular codon 72 polymorphism) to confer differences in cervical cancer risk [50].

Additionally, E6 variants R10G/L83V and Q14H/H78Y/L83V which are frequently detected in our study, were more prone to undergo cell-detachment-induced apoptosis than E6 prototype in a model of human normal immortalized keratinocytes (NIKS) [51] and has a greater in vitro ability to suppress keratinocyte differentiation responses and to induce p53 degradation than the prototype [52].

Interestingly, the E113D (A442C) was detected in 37.9% of samples and was exclusively found in the European variant (E350G/442C). This point mutation is largely reported in Asian populations and is implied in invasive cervical carcinoma [53-55].

The majorities of the E6 amino acid switches in our samples were located at amino acid residues10, 14, 78 and 83 and were shown to be under positive selective pressure [56,57]. This selection pressure, driven by protein–protein polymorphic interactions, could facilitate the life cycle and pathogenicity of HPV associated disease and might be under immune selection [57]. Most recently, Bayesian analysis identified several important amino acid positions that may be driving adaptive selection in the HPV 16 population, including R10G, L83V, and E113D in the E6 gene [48]. Therefore, the positive selection at these codons might be a contributing factor responsible for the phenotypic differences in carcinogenesis and immunogenicity among cervical cancers in Morocco. Indeed, it has been suggested that women with three distinct HLA class I alleles, namely HLA-B*44, HLA-B*51, or HLA-B*57 who were infected with the HPV16 E6 variant L83V had an approximately four to five fold increased risk for cancer compared with controls [49,58]. Additionally, in another study, the HPV 16 E6 variant G131 (R10G) was demonstrated to alter a B*07 binding epitope such that it may influence immune recognition by cytotoxic T lymphocytes [59]. Also, the immunologic relevance of the HPV16 E6 N-terminal region and variant positions E6 amino acids 10 and 14 is supported by the demonstration of an endogenously processed HLA A*0201-restricted E6 peptide (E6 amino acids 11 to 19) as well as of an overlapping HLA B-7-restricted E6 peptide (E6 amino acids 8 to 15) in this region [59,60].

Therefore, the observed amino acid variations in HPV16 E6 detected in our study may impact on the viral life cycle, which is tightly linked to the differentiation program of the cell [57].

## Conclusion

The introduction of molecular sub-typing of HPV allowed better genotyping and therefore a better characterization of strains circulating in Morocco. Thereby, the identification of HPV16 E, Af and NA1 variants suggests a different sexual mixing behavior. Their circulation in our population may involve a change in the biological properties and a change in the transforming potential, increasing the risk of cervical cancer development. Currently, Morocco is a destination of many sub-Saharan migrants looking for better life, and is also a gateway to Europe, that warrants the need for monitoring a wider range of HPV variants involved in cervical cancer in Morocco. Thus, in order to better understand the dynamics of the disease and to optimize current control programs and policies for a better survey of cervical cancer, it is useful to develop routine epidemiological surveillance, including molecular sub-typing.

## Competing interests

The authors declare that they have no competing interests.

## Authors’ contributions

ZQ: Carried out the molecular studies, participated in project design and drafted the manuscript; MK: participated in the conception and design of the study; MME: Participated in the conception and design of the study; MB: Specimen collection and data analysis; CN: Specimen collection and data analysis; LB: participated in the molecular analysis; NB: Sample collection, clinical data acquisition and anatomy pathology analyses; AB: Sample collection, clinical data acquisition and anatomy pathology analyses; MA: Conceived the study and participated in the design and coordination of the project and drafted the manuscript; MEM: Participated in the design and coordination of the project and review of the final manuscript. All authors read and approved the final manuscript.

## Authors’ information

Senior authors: Mohammed Attaleb and Mohammed El Mzibri.

## Pre-publication history

The pre-publication history for this paper can be accessed here:

http://www.biomedcentral.com/1471-2334/13/378/prepub
